# Serum uric acid levels in Parkinson's disease and related disorders

**DOI:** 10.1002/brb3.598

**Published:** 2016-10-31

**Authors:** Hideki Sakuta, Keisuke Suzuki, Tomoyuki Miyamoto, Masayuki Miyamoto, Ayaka Numao, Hiroaki Fujita, Yuji Watanabe, Koichi Hirata

**Affiliations:** ^1^ Department of NeurologyDokkyo Medical UniversityMibuJapan; ^2^ Department of NeurologyDokkyo Medical University Koshigaya HospitalKoshigayaJapan; ^3^ Department of Clinical Medicine for NursingDokkyo Medical University School of NursingMibuJapan

**Keywords:** multiple system atrophy, Parkinson's disease, progressive supranuclear palsy, uric acid

## Abstract

**Objective:**

Serum uric acid (UA) levels are reported to be decreased in patients with Parkinson's disease (PD) and multiple system atrophy (MSA). However, clinical correlates of serum UA levels are still unclear in PD‐related disorders. We conducted a cross‐sectional study to evaluate the associations between serum UA levels and disease duration, disease severity, and motor function among PD, MSA, and progressive supranuclear palsy (PSP) patients.

**Methods:**

A total of 100 patients with PD, 42 patients with MSA, 30 patients with PSP, and 100 controls were included in this study. Serum UA levels were determined, and associations among serum UA levels and disease duration, disease severity, and motor function in PD, PSP, and MSA patients were evaluated.

**Results:**

Serum UA levels were significantly lower in male PD, MSA, and PSP patients compared with the controls, but not in female patients. Serum UA levels were negatively correlated with disease duration and severity in MSA and PSP patients, but no correlations were observed in PD patients. The serum UA levels were significantly decreased in the tauopathy group (PSP patients) compared with the synucleinopathy group (PD and MSA patients) after adjusting for age, gender, and body mass index.

**Conclusion:**

We found decreased serum UA levels in male patients with PD‐related disorders (PD, MSA, and PSP) compared with male controls, and significant correlations between serum UA levels and disease severity in MSA and PSP patients.

## 1 Introduction

Serum uric acid (UA) levels have been reported to be lower in patients with Parkinson's disease (PD) and multiple system atrophy (MSA) than in control subjects (Annanmaki, Muuronen, & Murros, 2007; Constantinescu, Andreasson, Holmberg, & Zetterberg, 2013). Reactive oxygen species and oxidative stress may contribute to the pathogenesis of PD (Jenner & Olanow, 1996). Postmortem analyses of the brains of PD patients have reported increased iron levels as well as altered levels of other metal ions (Dexter et al., 1989). UA exerts antioxidant effects in neurons by acting as a scavenger of free radicals and as an iron chelator (Glantzounis, Tsimoyiannis, Kappas, & Galaris, 2005; Schlesinger & Schlesinger, 2008).

Moccia et al. (2015) found a relation between serum UA and dopamine transporter availability in drug‐naïve PD patients: serum UA levels were positively correlated with averaged, ipsilateral and contralateral dopamine transporter binding in the striatum, specifically in the caudate and putamen. In PD patients, low serum UA levels have been associated with cognitive dysfunction (Annanmaki, Pessala‐Driver, Hokkanen, & Murros, 2008). PD patients with the lowest quartiles of serum UA levels showed higher scores on the Unified PD Rating Scale part III (motor part), higher total nonmotor symptom (NMS) scores, and higher domain scores relating to sleep, mood, and gastrointestinal function (Pan et al., 2013). Moccia et al. (2014) reported that early‐stage, drug‐naïve PD patients with lower serum UA levels had higher scores in the attention/memory, cardiovascular, and sleep domains, as evaluated by NMSQuest. In MSA patients, no correlation has been found between serum UA levels at the initial visit and the mean rate of annual changes in the Unified MSA Rating Scale (Cao et al., 2013). However, few studies have compared serum UA levels among patients with PD‐related disorders, including PD, MSA, and progressive supranuclear palsy (PSP; Constantinescu et al., 2013). Recently, Oropesa‐Ruiz et al. (2016) have reported significantly reduced levels of serum UA in patients with PSP and PD compared with those in healthy controls. In their study, there was no correlation between disease duration and serum UA level in either PD or PSP patients.

We performed a cross‐sectional study to investigate the associations among the serum UA levels and background clinical factors in PD, PSP, and MSA patients.

## 2 Materials and Methods

**Figure 1:**
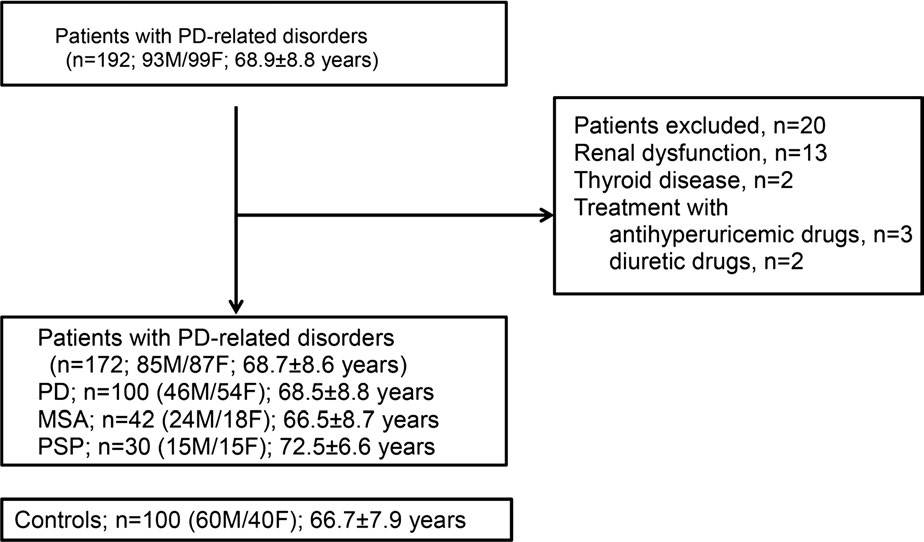
Flowchart of the patient selection process. PD, Parkinson's disease; MSA, multiple system atrophy; PSP, progressive supranuclear palsy

The present study was approved by the institutional review board of Dokkyo Medical University and conducted in accordance with the Declaration of Helsinki. All subjects provided written informed consent. This cross‐sectional study included 135 patients with parkinsonism (63 M/72 F; age, 68.6 ± 9.1 years) who were admitted to our hospital between April 2011 and April 2015. Figure 1 shows a flowchart of the patient selection process. The following patients were excluded from the study: those with renal dysfunction (estimated glomerular filtration rate <50 ml/min/1.73 m^2^), *n* = 13; those with thyroid disease, *n* = 2; those who were receiving treatment with antihyperuricemic drugs, *n* = 3; and those who were taking diuretics, *n* = 2. No patient had diabetic ketoacidosis, a body mass index (BMI) ≥30, and cancer or a hematological malignancy (e.g., leukemia or a myeloproliferative disease). The study included a total of 100 patients with PD (46 M/54 F; age, 68.7 ± 8.6 years), 42 with MSA (24 M/18 F; age, 66.5 ± 8.7 years), and 30 with PSP (15 M/15 F; age, 72.5 ± 6.6 years), as well as 100 healthy control subjects (60 M/40 F; age, 66.7 ± 7.9 years). The control subjects, who did not suffer from neurodegenerative or cerebrovascular diseases and met the above exclusion criteria were recruited from stable outpatients and medical staff in our hospital. A diagnosis of PD was made based on the UK PD Society Brain Bank clinical diagnostic criteria (Hughes, Daniel, Kilford, & Lees, 1992). MSA and PSP were diagnosed according to the second consensus statement on the diagnosis of MSA (Gilman et al., 2008) and the diagnostic criteria of the National Institute of Neurological Disorders and Stroke and the Society for PSP (Litvan et al., 1996), respectively. Disease severity was rated using Hoehn and Yahr (HY) staging (Hoehn & Yahr, 1967). The Unified PD Rating Scale (UPDRS) part III was used to assess motor symptoms. UPDRS part III has been reported to be a reliable assessment of motor symptoms in PSP (Cubo et al., 2000). In addition, for MSA patients, motor and cerebellar symptoms were assessed using the Unified MSA Rating Scale (UMSARS) part II.

Blood samples were collected from all the participants under nonfasting conditions and immediately analyzed following collection. Serum UA levels were determined in a clinical laboratory using standard clinical methods.

We investigated the correlations between serum UA levels and clinical background factors among PD, MSA, and PSP patients. In addition, we compared the serum UA levels of the combined PD and MSA group with those of the PSP group.

### 2.1 Statistical analyses

A Mann–Whitney *U* test or an unpaired *t* test was used, when appropriate, to compare continuous variables. A chi‐square or Fisher's exact test was used to compare categorical variables between the two groups. A general linear model was used to compare the estimated mean serum UA levels between the patient and control groups after adjusting for age and BMI. Correlations between the serum UA levels and other clinical parameters were analyzed using Spearman's rank correlation coefficients. Statistical significance was defined as a two‐tailed *p *< .05. GraphPad Prism for Windows (Version 5.01; GraphPad Software, San Diego, CA, USA) was used for the figures, and SPSS Statics 21.0 (IBM SPSS, Tokyo, Japan) was used for the statistical analyses.

## 3 Results

Table 1 lists the clinical characteristics of all the patients and controls. Age and BMI were significantly different among the groups. PD patients received higher levodopa‐equivalent doses (LEDs) than patients in the other groups. Among the clinical characteristics of male patients and controls, similar trends were found for age, BMI, and LED among the groups (Table 2). Serum UA levels (mean ± standard error) were significantly lower in PD (4.7 ± 1.2 mg/dl), MSA (4.6 ± 1.2 mg/dl) and PSP (4.0 ± 1.2 mg/dl) patients compared with the controls (5.5 ± 1.2 mg/dl) after adjusting for age, gender, and BMI (Table 3). In a gender‐specific analysis, serum UA levels were significantly decreased in male PD, MSA, and PSP patients relative to the controls, and serum UA levels were significantly lower in PSP patients than in PD patients. In contrast, no differences were observed in the serum UA levels of females among the groups (Table 3).

**Table 1:** Characteristics of all patients and controls

	PD	MSA	PSP	Controls	*p* value
*n* (M/F)	100 (46/54)	42 (24/18)	30 (15/15)	100 (60/40)	.091
Age (years)	68.5 ± 8.8	66.5 ± 8.7	72.5 ± 6.6	66.7 ± 7.9	.0056
BMI (kg/m^2^)	21.7 ± 3.2	23.2 ± 6.1	22.1 ± 4.2	24.5 ± 3.4	<.001
MMSE	25.7 ± 4.7	25.1 ± 5.6	23.4 ± 5.2	–	.14
Disease duration (years)	5.3 ± 5.3	4.9 ± 9.4	3.1 ± 2.4	–	.22
HY stage	3.1 ± 1.0	3.5 ± 1.2	3.5 ± 1.1	–	.12
UPDRS III	28.5 ± 14.2	29.5 ± 21.0	34.3 ± 21.0	1.8 ± 1.9	<.001
UMSARS II	–	22.6 ± 12.5	–	–	–
Drug‐naïve patients, *n* (%)	48 (48.0)	27 (64.3)	23 (76.7)	–	0.011
LED (mg/day)	363.9 ± 424.6	100.0 ± 168.2	116.0 ± 214.5	–	<0.001

BMI, body mass index; HY, Hoehn and Yahr; PD, Parkinson's disease; MSA, multiple system atrophy; PSP, progressive supranuclear palsy; MMSE, Mini‐Mental State Examination; UPDRS, Unified PD Rating Scale; UMSARS, Unified MSA Rating Scale; LED, levodopa‐equivalent dose.

**Table 2:** Characteristics of male patients and controls

	PD	MSA	PSP	Controls	*p* value
*n*	46	24	15	60	
Age (years)	68.7 ± 8.3	68.1 ± 8.0	71.4 ± 7.5	65.6 ± 8.5	.060
BMI (kg/m^2^)	22.3 ± 3.0	22.9 ± 2.9	21.3 ± 4.2	24.9 ± 2.6	<.001
MMSE	25.1 ± 5.2	25.2 ± 4.4	23.1 ± 6.4	–	.66
Disease duration (years)	4.7 ± 3.9	3.1 ± 2.4	3.4 ± 2.5	–	.10
HY stage	3.0 ± 1.0	3.2 ± 1.1	3.7 ± 1.2	–	.055
UPDRS III	29.0 ± 14.8	28.1 ± 19.3	36.0 ± 26.0	1.6 ± 1.8	<.001
UMSARS II	–	18.8 ± 11.9	–	–	
Drug‐naïve patients, *n* (%)	19 (41.3)	17 (70.8)	13 (86.7)	–	<.001
LED (mg/day)	281.8 ± 302.3	58.3 ± 121.3	110.7 ± 222.0	–	<.001

BMI, body mass index; HY, Hoehn and Yahr; PD, Parkinson's disease; MSA, multiple system atrophy; PSP, progressive supranuclear palsy; MMSE, Mini‐Mental State Examination; UPDRS, Unified PD Rating Scale; UMSARS, Unified MSA Rating Scale; LED, levodopa‐equivalent dose.

**Table 3:** Serum UA level differences among PD, MSA, and PSP patients and controls

	Controls	PD	MSA	PSP
Serum UA levels (mg/dl)				
Total^a^	5.5 ± 1.2^c^	4.7 ± 1.2	4.6 ± 1.2	4.0 ± 1.2
Males^b^ (*n*)	6.0 ± 1.2^c^	4.9 ± 1.1	5.0 ± 1.1	4.0 ± 1.2^d^
Females^b^ (*n*)	4.7 ± 1.2	4.4 ± 1.2	4.2 ± 1.2	4.1 ± 1.2

PD, Parkinson's disease; MSA, multiple system atrophy; PSP, progressive supranuclear palsy; UA, uric acid.

Data are represented as the mean ± standard error.

a Adjusted for age, sex, and BMI.

b Adjusted for age and BMI.

c *p *< .05 compared with PD, MSA, and PSP.

d *p *< .05 compared with PD.

When the patients were classified into the combined PD and MSA group (*n* = 142) and the PSP group (*n* = 30), the serum UA levels were significantly decreased in the PSP group compared with the PD and MSA group after adjusting for age, gender, and BMI (4.0 ± 1.4 mg/dl vs. 4.6 ± 1.2 mg/dl, *p *< .001). In a gender‐specific analysis, this significant difference was observed in male patients, but not in female patients.

Table 4 shows the correlation analyses between serum UA levels and clinical parameters. The serum UA levels were negatively correlated with disease duration, HY stage, and UMSARS part II in MSA patients and with disease duration, HY stage, and UPDRS part III in PSP patients. No significant correlations of the serum UA levels with these parameters were observed in PD patients. A positive correlation was found between age at onset and serum UA levels only in female MSA patients. BMI and the serum UA levels were positively correlated in the all MSA and PSP patients, in the control subjects, and in the male PD patients.

**Table 4:** Correlation coefficients between serum UA and other clinical factors in patients and controls

	Serum UA											
	PD			MSA			PSP			Controls		
	Total	Men	Women	Total	Men	Women	Total	Men	Women	Total	Men	Women
Age	−0.039	−0.049	−0.040	0.150	−0.19	0.43	0.14	0.15	0.096	−0.44	0.027	0.12
BMI	0.19	0.30*	0.024	0.49**	0.17	0.75**	0.57**	0.66**	0.56*	0.27*	0.23	0.19
MMSE	−0.080	0.22	−0.13	−0.23	−0.14	−0.26	0.13	0.13	0.017	–	–	–
Disease duration	0.054	0.20	−0.038	−0.31*	−0.34	−0.18	−0.37*	−0.70**	−0.011	–	–	–
HY stage	−0.085	−0.24	0.11	−0.39*	−0.24	−0.44	−0.44*	−0.59*	−0.23	–	–	–
UPDRS III	−0.010	−0.18	0.11	−0.24	−0.35	−0.11	−0.49**	−0.62*	−0.095	0.063	–	–
UMSARS II	–	–	–	−0.32*	−0.34	−0.28	–	–	–	–	–	–
LED	−0.015	0.020	0.020	−0.21	−0.003	−0.25	−0.36	−0.26	−0.54*	–	–	–

UA, uric acid; BMI, body mass index; HY, Hoehn and Yahr; PD, Parkinson's disease; MSA, multiple system atrophy; PSP, progressive supranuclear palsy; MMSE, Mini‐Mental State Examination; UPDRS, Unified PD Rating Scale; UMSARS, Unified MSA Rating Scale; LED, levodopa‐equivalent dose.

*p < .05, **p < .01, ***p < .001; Spearman's rank correlation.

## 4 Discussion

We found that serum UA levels were lower in male PD, MSA, and PSP patients than in male control subjects, but this relationship was not observed in females. This difference between men and women may be related to the different influence of estrogen on UA metabolism in women (Gillies & McArthur, 2010) and gender differences in the renal handling of UA, regardless of the influence of estradiol (Anton, Garcia Puig, Ramos, Gonzalez, & Ordas, 1986). Serum UA levels are generally higher in men than in women. Similarly, previous studies have found reduced serum UA levels in male PD and MSA patients compared with male control subjects (Cao et al., 2013; Constantinescu et al., 2013), but these studies did not include PSP patients.

Constantinescu et al. (2013) classified parkinsonian patients into synucleinopathy and tauopathy groups and not only observed lower serum UA levels in patients with synucleinopathy than in those with tauopathy, but also observed no difference in cerebrospinal fluid UA levels. By contrast, in our study, the serum UA levels were significantly decreased in the tauopathy group (PSP) compared with the synucleinopathy group (PD and MSA). In accordance with our results, a recent study including 47 PSP patients and 225 PD patients showed that serum UA levels tended to be lower in PSP than in PD (Oropesa‐Ruiz et al., 2016). Decreased glutathione has been observed in the substantia nigra and other brain regions of PD and PSP patients (Fitzmaurice et al., 2003), which could result in abnormal UA utilization and may have a role in the decreased serum UA levels in these disorders.

In our study, there was no significant correlation between UA levels and disease severity in PD patients. A recent study found no correlation between UA levels and disease severity in untreated PD patients, but found that lower UA levels were significantly associated with lower dopamine transporter binding in the caudate, putamen, and striatum (Moccia et al., 2015). Meamar, Shaabani, Tabibian, Aghaye Ghazvini, and Feizi (2015) observed positive but statistically insignificant correlations of serum UA levels with HY stage and UPDRS part III in PD patients using regression models and found a positive relationship between serum UA levels and UPDRS part III in PD patients aged 62 years or younger.

In contrast, we observed significant inverse correlations between UA levels, disease severity, and motor function in MSA and PSP patients. Sun et al. (2012) observed an inverse correlation between UA levels and disease severity in PD patients, but other studies have not (Andreadou et al., 2009). A recent cross‐sectional study found that decreased serum UA levels predicted the development of wearing‐off in patients with PD (Fukae et al., 2014). To the best of our knowledge, no studies have performed a cross‐sectional evaluation of the correlation between serum UA levels and motor function in PSP and MSA patients; our study demonstrated the negative correlations between serum UA levels and motor function in MSA and PSP patients for the first time.

Based on the case–control studies, subjects with increased levels of serum UA and gout were associated with a decreased risk for developing PD (Weisskopf, O'Reilly, Chen, Schwarzschild, & Ascherio, 2007), although in one study, the plasma UA levels in women were not significantly associated with the risk of PD (O'Reilly et al., 2010). Based on a prospective population study, higher levels of serum UA were also associated with a decreased risk of PD (Chen, Mosley, Alonso, & Huang, 2009; de Lau, Koudstaal, Hofman, & Breteler, 2005). Low serum UA levels have been associated with an increased risk for developing PD, but it is unclear whether the UA levels are generally low among individuals who are prone to PD or whether the UA levels decrease during the long preclinical stages of PD (Annanmaki, Pohja, Parviainen, Hakkinen, & Murros, 2011). Several studies suggest that variants or polymorphisms of the UA transporter gene may delay the development of PD. Dysfunctional variants of ATP‐binding cassette transporter, sub‐family G, member 2 (ABCG2) causing elevated serum UA levels have been reported to be associated not only with the early onset of gout but also with a later onset of PD (Matsuo et al., 2015). In addition, variations in the UA transporter gene SLC2A9 associated with lower serum UA levels have been found to be associated with the onset of PD at a younger age (Facheris et al., 2011). However, in our study, a significant correlation between age at onset and serum UA levels was only evident among female patients with MSA. Our observations of the lack of a correlation between the serum UA levels and disease severity in PD but an inverse correlation in MSA and PSP patients may reflect the longer prodromal phase of PD compared with MSA and PSP.

Increased serum UA levels have been associated with gout, hypertension, and cardiovascular disease, and decreased serum UA levels have been associated with PD (Schlesinger & Schlesinger, 2008). Thus, UA may play a different role in patients than in healthy individuals. In contrast, in drug‐naïve PD patients, lower UA levels have been associated with cardiovascular domains, as evaluated by NMSQuest (Moccia et al., 2014). Pan et al. (2013) also found a trend of higher cardiovascular scores of NMS domains in PD patients in the lowest serum UA level quartile compared with PD patients in the highest serum UA level quartile. However, it should be noted that the cardiovascular domain items of NMSQuest were “feeling light headed, dizzy, or weak standing from sitting or lying,” and “falling in the last month,” which are different from typical symptoms due to cardiovascular diseases.

The limitations of this study include the cross‐sectional design, which made it difficult to evaluate whether serum UA levels changed over the course of the disease. A significant number of patients were treated with dopaminergic drugs. Levodopa decreases UA excretion via renal tubular transport and increases serum UA levels (Schlesinger & Schlesinger, 2008); therefore, the reduced serum UA levels observed in patients with PD‐related disorders compared with the controls were likely not due to the effects of dopaminergic treatments. However, differences in LEDs between PD and PSP patients may have influenced the decreased serum UA levels in PSP patients compared with PD patients. Additionally, the serum UA levels were measured in the nonfasting state, and dietary factors were not considered; however, in a previous study, lower plasma UA levels were reported in PD patients than in controls, despite equivalent daily caloric consumption in the two groups (Annanmaki et al., 2007).

In conclusion, relative to control subjects, patients with PD‐related disorders exhibited decreased serum UA levels, and significant correlations between serum UA levels and both disease duration and disease severity were observed in MSA and PSP patients.

## Conflict of Interest

The authors declare that they have no conflicts of interest.
